# (Carbonato-κ^2^ *O*,*O*′)bis­(1,10-phenan­throline-κ^2^ *N*,*N*′)cobalt(III) nitrate monohydrate

**DOI:** 10.1107/S1600536809052763

**Published:** 2009-12-12

**Authors:** Ömer Andaç, Zuhal Yolcu, Orhan Büyükgüngör

**Affiliations:** a Ondokuzmayıs Üniversitesi, Fen-Edebiyat Faültesi, Kimya Bölümü, 55200 Atakum, Samsun, Turkey; b Ondokuzmayıs Üniversitesi, Fen-Edebiyat Faültesi, Fizik Bölümü, 55200 Atakum, Samsun, Turkey

## Abstract

The crystal structure of the title compound, [Co(CO_3_)(C_12_H_8_N_2_)_2_]NO_3_·H_2_O, consists of Co^III^ complex cations, nitrate anions and uncoordinated water mol­ecules. The Co^III^ cation is chelated by a carbonate anion and two phenanthroline ligands in a distorted octa­hedral coordination geometry. A three-dimensional supra­molecular structure is formed by O—H⋯O and C—H⋯O hydrogen bonding, C—H⋯π and aromatic π–π stacking [centroid–centroid distance = 3.995 (1)Å] inter­actions.

## Related literature

For Co(III) complexes with carbonate and phen ligands, see: Fu *et al.* (2006 ▶); Guild *et al.* (1980 ▶); Hadadzadeh *et al.* (2007 ▶); Hennig *et al.* (1980 ▶); McAuliffe *et al.* (1992 ▶); Niederhoffer *et al.* (1982 ▶); Sharma *et al.* (2009 ▶). For a Co^II^ coordination compound with carbonate and phen ligands, see: Li *et al.* (2004 ▶).
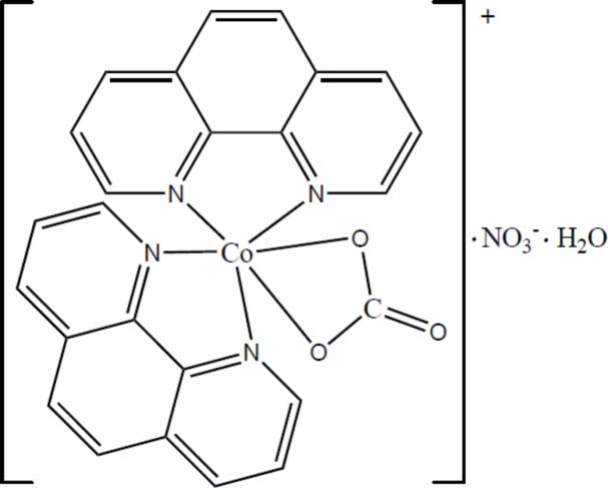


## Experimental

### 

#### Crystal data

[Co(CO_3_)(C_12_H_8_N_2_)_2_]NO_3_·H_2_O
*M* *_r_* = 559.37
Monoclinic, 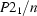

*a* = 13.6986 (9) Å
*b* = 10.8583 (5) Å
*c* = 16.1494 (10) Å
β = 106.386 (5)°
*V* = 2304.6 (2) Å^3^
*Z* = 4
Mo *K*α radiation
μ = 0.80 mm^−1^
*T* = 296 K
0.41 × 0.26 × 0.15 mm

#### Data collection

Stoe IPDS-2 diffractometer
Absorption correction: integration (*X-RED32*; Stoe & Cie, 2002 ▶) *T*_min_ = 0.818, *T*_max_ = 0.905
21353 measured reflections
5713 independent reflections
4021 reflections with *I* > 2σ(*I*)
*R*_int_ = 0.045

#### Refinement

*R*[*F* ^2^ > 2σ(*F* ^2^)] = 0.036
*wR*(*F* ^2^) = 0.102
*S* = 1.02
5713 reflections
349 parameters
3 restraints
H atoms treated by a mixture of independent and constrained refinement
Δρ_max_ = 0.26 e Å^−3^
Δρ_min_ = −0.42 e Å^−3^

### 

Data collection: *X-AREA* (Stoe & Cie, 2002 ▶); cell refinement: *X-AREA*; data reduction: *X-RED32* (Stoe & Cie, 2002 ▶); program(s) used to solve structure: *SHELXS97* (Sheldrick, 2008 ▶); program(s) used to refine structure: *SHELXL97* (Sheldrick, 2008 ▶); molecular graphics: *ORTEP-3 for Windows* (Farrugia, 1997 ▶); software used to prepare material for publication: *SHELXL97*.

## Supplementary Material

Crystal structure: contains datablocks I, global. DOI: 10.1107/S1600536809052763/xu2707sup1.cif

Structure factors: contains datablocks I. DOI: 10.1107/S1600536809052763/xu2707Isup2.hkl

Additional supplementary materials:  crystallographic information; 3D view; checkCIF report

## Figures and Tables

**Table 1:** Hydrogen-bond geometry (Å, °)

*D*—H⋯*A*	*D*—H	H⋯*A*	*D*⋯*A*	*D*—H⋯*A*
O7—H7*A*⋯O5	0.86 (2)	1.98 (2)	2.830 (3)	168 (5)
O7—H7*B*⋯O3^i^	0.81 (5)	2.03 (5)	2.809 (4)	164 (6)
C3—H3⋯O3^ii^	0.93	2.54	3.362 (3)	148
C5—H5⋯O1^ii^	0.93	2.56	3.415 (3)	153
C8—H8⋯O7^iii^	0.93	2.31	3.130 (4)	146
C9—H9⋯O6^iv^	0.93	2.35	3.234 (3)	158
C15—H15⋯O6^v^	0.93	2.45	3.370 (3)	170
C17—H17⋯O4^v^	0.93	2.50	3.416 (3)	167
C1—H1⋯*Cg*1^vi^	0.93	2.92	3.705 (3)	143

Symmetry codes: (i) 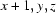; (ii) 
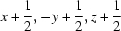
; (iii) 
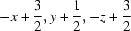
; (iv) 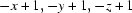; (v) 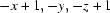; (vi) 
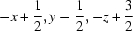
.

**Fig. 1.**
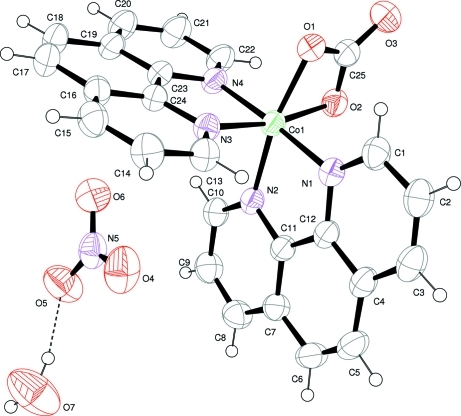
An ORTEP-III drawing of title complex with the atom numbering scheme at 40% ellipsoid.

**Fig. 2.**
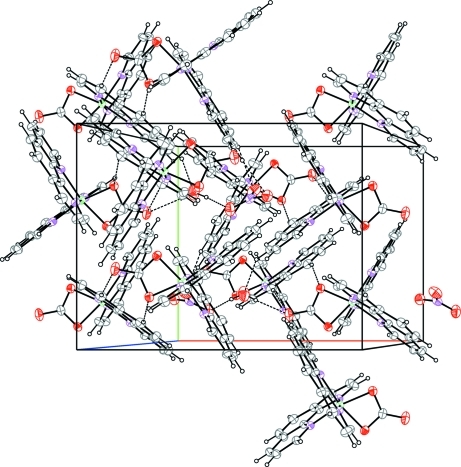
The packing diagram of the complex with hydrogen bonds shown as dashed lines.

## supplementary crystallographic information

### Comment

The stucture of the title compound, (I), is shown below. Dimensions are available in the archived CIF. The combination of cobalt(II) cations, carbonate and phenantroline ligands often results in Co^III^-containing products as a result of an autoxidation reaction. CCDC search revealed that several crystal structures of Co^III^ mixed ligand complexes of phenanthroline and carbonate ligands have been reported (Guild *et al.*, 1980; Fu *et al.*, 2006; Hadadzadeh *et al.*, 2007; McAuliffe *et al.*,1992; Hennig *et al.*, 1980; Niederhoffer *et al.*, 1982; Sharma *et al.*, 2009). Co^II^ coordination compound with carbonate and phen ligand has also been reported (Li *et al.*, 2004).

In this study, we describe the synthesis and structure of the title compound, (I). The Co^III^ atom in the discrete [Co(CO_3_)(phen)_2_]^+^ cation (Fig. 1) displays a distorted octahedral geometry, being coordinated by four N atoms of two 1,10-phenanthroline ligands and two O atoms of the bidentate carbonate anion. Both phen ligands are planar with r.m.s. deviation of 0.04 Å and involve in π–π interactions with neighboring phen rings. The carbonate ligand is also planar with r.m.s. deviation of 0.002 Å. The charge-balancing nitrate ion is essencially planar with r.m.s. deviation of 0.0015 Å and exibits slight deviation from D_3h_ symmetry. Non-coordinated water molecule is involved in hydrogen bonding (Table 1) and contrubutes stabilization of the 3 d structure forming. As shown in Fig. 2, the hydrogen bonds are supplemented by aromatic π–π stacking interactions of neighboring phen rings, C–H···O and C–H···π interactions.

### Experimental

Co(NO_3_)_2_.6H_2_O (0.291 g, 1.0 mmol) was added to a solution containing phen (0.396 g, 2.0 mmol) and adenine (0.135 g, 1.0 mmol) in water-ethanol (10:90, 100 ml). The reaction mixture was stirred for 1 h at 343 K. Thereafter, NaHCO_3_ solution (0.168 g, 2.00 mmol in 10 ml water-ethanole, 10:90) was added to the mixture for adjusting the pH to 7 by using solid-state pH sensor. The resulting solution was cooled to room temperature and filtered. The filtrate was left to stand in air for slow evaporation and red prism single crystals of (I) were obtained after several months.

### Refinement

Water H atoms were located in a difference Fourier map and refined with distance constraints of O—H = 0.83 (3) Å, U_iso_(H) = 1.5U_eq_(O). Other H atoms were placed in calculated positions with C—H = 0.93 Å, and refined in riding mode with U_iso_(H) = 1.2U_eq_(C).

### Crystal data

**Table:**

[Co(CO_3_)(C_12_H_8_N_2_)_2_]NO_3_·H_2_O	*F*(000) = 1144
*M**_r_* = 559.37	*D*_x_ = 1.612 Mg m^−^^3^
Monoclinic, *P*2_1_/*n*	Mo *K*α radiation, λ = 0.71073 Å
Hall symbol: -P 2yn	Cell parameters from 23029 reflections
*a* = 13.6986 (9) Å	θ = 1.6–28.4°
*b* = 10.8583 (5) Å	µ = 0.80 mm^−^^1^
*c* = 16.1494 (10) Å	*T* = 296 K
β = 106.386 (5)°	Prism, red
*V* = 2304.6 (2) Å^3^	0.41 × 0.26 × 0.15 mm
*Z* = 4	

### Data collection

**Table:**

Stoe IPDS-2 diffractometer	5713 independent reflections
Radiation source: fine-focus sealed tube	4021 reflections with *I* > 2σ(*I*)
graphite	*R*_int_ = 0.045
Detector resolution: 6.67 pixels mm^-1^	θ_max_ = 28.3°, θ_min_ = 1.7°
rotation method scans	*h* = −18→18
Absorption correction: integration (*X-RED32*; Stoe & Cie, 2002)	*k* = −14→14
*T*_min_ = 0.818, *T*_max_ = 0.905	*l* = −21→21
21353 measured reflections	

### Refinement

**Table:**

Refinement on *F*^2^	Primary atom site location: structure-invariant direct methods
Least-squares matrix: full	Secondary atom site location: difference Fourier map
*R*[*F*^2^ > 2σ(*F*^2^)] = 0.036	Hydrogen site location: inferred from neighbouring sites
*wR*(*F*^2^) = 0.102	H atoms treated by a mixture of independent and constrained refinement
*S* = 1.02	*w* = 1/[σ^2^(*F*_o_^2^) + (0.0554*P*)^2^ + 0.0979*P*] where *P* = (*F*_o_^2^ + 2*F*_c_^2^)/3
5713 reflections	(Δ/σ)_max_ = 0.001
349 parameters	Δρ_max_ = 0.26 e Å^−^^3^
3 restraints	Δρ_min_ = −0.42 e Å^−^^3^

### Special details

**Table:**

Geometry. All e.s.d.'s (except the e.s.d. in the dihedral angle between two l.s. planes) are estimated using the full covariance matrix. The cell e.s.d.'s are taken into account individually in the estimation of e.s.d.'s in distances, angles and torsion angles; correlations between e.s.d.'s in cell parameters are only used when they are defined by crystal symmetry. An approximate (isotropic) treatment of cell e.s.d.'s is used for estimating e.s.d.'s involving l.s. planes.
Refinement. Refinement of *F*^2^ against ALL reflections. The weighted *R*-factor *wR* and goodness of fit *S* are based on *F*^2^, conventional *R*-factors *R* are based on *F*, with *F* set to zero for negative *F*^2^. The threshold expression of *F*^2^ > σ(*F*^2^) is used only for calculating *R*-factors(gt) *etc*. and is not relevant to the choice of reflections for refinement. *R*-factors based on *F*^2^ are statistically about twice as large as those based on *F*, and *R*- factors based on ALL data will be even larger.

### Fractional atomic coordinates and isotropic or equivalent isotropic displacement parameters (Å^2^)

**Table:**

	*x*	*y*	*z*	*U*_iso_*/*U*_eq_	
C1	0.23394 (19)	0.2180 (2)	0.81209 (14)	0.0555 (5)	
H1	0.1733	0.1780	0.7850	0.067*	
C2	0.2717 (2)	0.2124 (2)	0.90169 (16)	0.0634 (6)	
H2	0.2351	0.1713	0.9337	0.076*	
C3	0.3620 (2)	0.2669 (2)	0.94241 (15)	0.0622 (6)	
H3	0.3881	0.2607	1.0020	0.075*	
C4	0.41549 (18)	0.3325 (2)	0.89410 (13)	0.0517 (5)	
C5	0.50875 (19)	0.3963 (2)	0.92853 (14)	0.0614 (6)	
H5	0.5413	0.3911	0.9873	0.074*	
C6	0.55098 (18)	0.4642 (2)	0.87781 (15)	0.0614 (6)	
H6	0.6106	0.5076	0.9026	0.074*	
C7	0.50573 (17)	0.4708 (2)	0.78621 (14)	0.0519 (5)	
C8	0.54379 (19)	0.5401 (2)	0.72896 (17)	0.0621 (6)	
H8	0.6029	0.5862	0.7493	0.074*	
C9	0.49337 (19)	0.5391 (2)	0.64327 (16)	0.0602 (6)	
H9	0.5175	0.5858	0.6050	0.072*	
C10	0.40593 (17)	0.4685 (2)	0.61276 (14)	0.0513 (5)	
H10	0.3734	0.4677	0.5538	0.062*	
C11	0.41689 (15)	0.40448 (19)	0.75099 (12)	0.0446 (4)	
C12	0.37090 (16)	0.33652 (19)	0.80467 (12)	0.0452 (4)	
C13	0.36955 (17)	0.0689 (2)	0.68057 (14)	0.0530 (5)	
H13	0.3751	0.0805	0.7388	0.064*	
C14	0.41500 (19)	−0.0337 (2)	0.65606 (16)	0.0614 (6)	
H14	0.4503	−0.0894	0.6976	0.074*	
C15	0.40802 (19)	−0.0530 (2)	0.57096 (17)	0.0604 (6)	
H15	0.4380	−0.1220	0.5542	0.072*	
C16	0.35541 (17)	0.0319 (2)	0.50914 (14)	0.0522 (5)	
C17	0.3447 (2)	0.0247 (2)	0.41841 (16)	0.0623 (6)	
H17	0.3719	−0.0425	0.3969	0.075*	
C18	0.2962 (2)	0.1128 (2)	0.36378 (15)	0.0631 (6)	
H18	0.2919	0.1062	0.3054	0.076*	
C19	0.25118 (18)	0.2164 (2)	0.39328 (14)	0.0522 (5)	
C20	0.1959 (2)	0.3097 (2)	0.34048 (15)	0.0651 (6)	
H20	0.1888	0.3093	0.2815	0.078*	
C21	0.1521 (2)	0.4017 (3)	0.37623 (15)	0.0675 (7)	
H21	0.1144	0.4631	0.3414	0.081*	
C22	0.16433 (19)	0.4027 (2)	0.46493 (14)	0.0570 (5)	
H22	0.1343	0.4654	0.4884	0.068*	
C23	0.25937 (16)	0.22439 (18)	0.48130 (13)	0.0458 (4)	
C24	0.31266 (15)	0.13283 (19)	0.53936 (12)	0.0447 (4)	
C25	0.07886 (18)	0.3405 (2)	0.63398 (14)	0.0563 (5)	
N5	0.57159 (19)	0.29524 (19)	0.57205 (15)	0.0663 (6)	
N1	0.28239 (14)	0.27885 (15)	0.76449 (11)	0.0460 (4)	
N2	0.36749 (13)	0.40223 (15)	0.66510 (10)	0.0434 (4)	
N3	0.31838 (13)	0.15092 (16)	0.62389 (10)	0.0450 (4)	
N4	0.21773 (13)	0.31645 (16)	0.51679 (10)	0.0464 (4)	
O1	0.11556 (12)	0.23030 (15)	0.62705 (10)	0.0545 (4)	
O2	0.15083 (11)	0.42331 (14)	0.64563 (9)	0.0528 (4)	
O3	−0.00973 (13)	0.3624 (2)	0.63088 (13)	0.0822 (6)	
O4	0.5792 (2)	0.2517 (2)	0.64330 (14)	0.0981 (7)	
O5	0.64324 (16)	0.3479 (3)	0.55465 (15)	0.0973 (7)	
O6	0.48923 (17)	0.2860 (2)	0.51406 (14)	0.0837 (6)	
O7	0.8229 (2)	0.2576 (4)	0.6729 (2)	0.1344 (11)	
H7A	0.764 (2)	0.284 (5)	0.643 (3)	0.202*	
H7B	0.863 (3)	0.289 (5)	0.651 (4)	0.202*	
Co1	0.24569 (2)	0.29966 (2)	0.640409 (16)	0.04249 (9)	

### Atomic displacement parameters (Å^2^)

**Table:**

	*U*^11^	*U*^22^	*U*^33^	*U*^12^	*U*^13^	*U*^23^
C1	0.0660 (14)	0.0558 (13)	0.0496 (12)	−0.0034 (10)	0.0241 (11)	0.0011 (9)
C2	0.0807 (17)	0.0649 (15)	0.0516 (13)	0.0025 (13)	0.0299 (13)	0.0099 (11)
C3	0.0823 (18)	0.0644 (15)	0.0393 (11)	0.0122 (13)	0.0163 (12)	0.0072 (10)
C4	0.0601 (13)	0.0547 (12)	0.0383 (10)	0.0106 (10)	0.0108 (9)	−0.0004 (9)
C5	0.0639 (14)	0.0688 (15)	0.0420 (11)	0.0113 (12)	−0.0006 (10)	−0.0074 (10)
C6	0.0499 (13)	0.0686 (15)	0.0570 (13)	0.0003 (11)	0.0008 (11)	−0.0126 (11)
C7	0.0487 (12)	0.0522 (11)	0.0531 (11)	0.0026 (10)	0.0117 (10)	−0.0065 (9)
C8	0.0556 (13)	0.0582 (13)	0.0737 (16)	−0.0091 (11)	0.0206 (12)	−0.0069 (12)
C9	0.0657 (15)	0.0568 (13)	0.0637 (14)	−0.0037 (11)	0.0274 (12)	0.0056 (11)
C10	0.0575 (13)	0.0523 (12)	0.0475 (11)	0.0017 (10)	0.0203 (10)	0.0024 (9)
C11	0.0455 (10)	0.0467 (10)	0.0404 (10)	0.0039 (9)	0.0101 (8)	−0.0026 (8)
C12	0.0510 (12)	0.0463 (10)	0.0373 (9)	0.0059 (9)	0.0109 (9)	−0.0014 (8)
C13	0.0607 (13)	0.0512 (11)	0.0486 (11)	0.0065 (10)	0.0180 (10)	0.0064 (9)
C14	0.0679 (15)	0.0517 (12)	0.0663 (14)	0.0127 (11)	0.0216 (12)	0.0101 (11)
C15	0.0640 (14)	0.0489 (12)	0.0729 (15)	0.0079 (11)	0.0269 (12)	−0.0029 (11)
C16	0.0529 (12)	0.0516 (12)	0.0561 (12)	0.0000 (10)	0.0220 (10)	−0.0062 (10)
C17	0.0688 (15)	0.0632 (14)	0.0605 (14)	0.0023 (12)	0.0273 (12)	−0.0154 (12)
C18	0.0727 (15)	0.0740 (16)	0.0475 (12)	−0.0024 (13)	0.0253 (12)	−0.0130 (11)
C19	0.0563 (12)	0.0597 (13)	0.0401 (10)	−0.0035 (10)	0.0126 (9)	−0.0060 (9)
C20	0.0786 (17)	0.0763 (16)	0.0381 (11)	0.0016 (14)	0.0126 (11)	0.0002 (11)
C21	0.0809 (17)	0.0702 (15)	0.0446 (12)	0.0139 (14)	0.0065 (12)	0.0069 (11)
C22	0.0643 (14)	0.0577 (13)	0.0431 (11)	0.0109 (11)	0.0054 (10)	0.0003 (10)
C23	0.0470 (11)	0.0506 (11)	0.0391 (10)	−0.0018 (9)	0.0112 (8)	−0.0049 (8)
C24	0.0453 (11)	0.0479 (11)	0.0421 (10)	−0.0015 (9)	0.0142 (9)	−0.0031 (8)
C25	0.0484 (12)	0.0756 (15)	0.0433 (11)	0.0041 (11)	0.0104 (9)	−0.0088 (10)
N5	0.0804 (16)	0.0622 (12)	0.0615 (12)	0.0192 (12)	0.0284 (12)	0.0051 (10)
N1	0.0511 (10)	0.0473 (9)	0.0407 (8)	0.0017 (7)	0.0148 (8)	−0.0011 (7)
N2	0.0482 (9)	0.0455 (8)	0.0373 (8)	0.0039 (7)	0.0131 (7)	−0.0007 (7)
N3	0.0481 (9)	0.0459 (9)	0.0415 (8)	0.0012 (8)	0.0134 (7)	0.0008 (7)
N4	0.0495 (10)	0.0496 (9)	0.0380 (8)	0.0045 (8)	0.0086 (7)	−0.0035 (7)
O1	0.0517 (8)	0.0618 (9)	0.0496 (8)	−0.0056 (7)	0.0135 (7)	−0.0104 (7)
O2	0.0521 (8)	0.0571 (9)	0.0488 (8)	0.0060 (7)	0.0136 (7)	−0.0076 (7)
O3	0.0494 (10)	0.1140 (16)	0.0828 (13)	0.0074 (10)	0.0181 (9)	−0.0190 (12)
O4	0.130 (2)	0.1037 (15)	0.0671 (13)	0.0381 (15)	0.0386 (13)	0.0315 (12)
O5	0.0698 (13)	0.1280 (19)	0.0965 (16)	0.0040 (13)	0.0278 (12)	0.0277 (14)
O6	0.0788 (13)	0.0934 (15)	0.0770 (13)	0.0005 (11)	0.0190 (11)	−0.0041 (11)
O7	0.0868 (17)	0.166 (3)	0.151 (3)	0.030 (2)	0.0343 (19)	0.085 (2)
Co1	0.04562 (16)	0.04603 (15)	0.03554 (14)	0.00165 (12)	0.01098 (11)	−0.00324 (11)

### Geometric parameters (Å, °)

**Table:**

C1—N1	1.325 (3)	C16—C17	1.433 (3)
C1—C2	1.394 (3)	C17—C18	1.343 (4)
C1—H1	0.9300	C17—H17	0.9300
C2—C3	1.361 (4)	C18—C19	1.428 (3)
C2—H2	0.9300	C18—H18	0.9300
C3—C4	1.406 (3)	C19—C23	1.397 (3)
C3—H3	0.9300	C19—C20	1.401 (3)
C4—C12	1.401 (3)	C20—C21	1.373 (4)
C4—C5	1.421 (3)	C20—H20	0.9300
C5—C6	1.348 (4)	C21—C22	1.394 (3)
C5—H5	0.9300	C21—H21	0.9300
C6—C7	1.435 (3)	C22—N4	1.330 (3)
C6—H6	0.9300	C22—H22	0.9300
C7—C11	1.390 (3)	C23—N4	1.356 (3)
C7—C8	1.401 (3)	C23—C24	1.419 (3)
C8—C9	1.361 (3)	C24—N3	1.359 (2)
C8—H8	0.9300	C25—O3	1.224 (3)
C9—C10	1.390 (3)	C25—O2	1.308 (3)
C9—H9	0.9300	C25—O1	1.315 (3)
C10—N2	1.327 (3)	C25—Co1	2.301 (2)
C10—H10	0.9300	N5—O4	1.221 (3)
C11—N2	1.361 (2)	N5—O5	1.235 (3)
C11—C12	1.415 (3)	N5—O6	1.251 (3)
C12—N1	1.356 (3)	N1—Co1	1.9367 (17)
C13—N3	1.327 (3)	N2—Co1	1.9513 (17)
C13—C14	1.387 (3)	N3—Co1	1.9548 (17)
C13—H13	0.9300	N4—Co1	1.9324 (16)
C14—C15	1.367 (3)	O1—Co1	1.8907 (16)
C14—H14	0.9300	O2—Co1	1.8869 (15)
C15—C16	1.400 (3)	O7—H7A	0.86 (2)
C15—H15	0.9300	O7—H7B	0.81 (5)
C16—C24	1.394 (3)		
			
N1—C1—C2	121.6 (2)	C19—C20—H20	120.2
N1—C1—H1	119.2	C20—C21—C22	119.8 (2)
C2—C1—H1	119.2	C20—C21—H21	120.1
C3—C2—C1	120.2 (2)	C22—C21—H21	120.1
C3—C2—H2	119.9	N4—C22—C21	121.8 (2)
C1—C2—H2	119.9	N4—C22—H22	119.1
C2—C3—C4	119.8 (2)	C21—C22—H22	119.1
C2—C3—H3	120.1	N4—C23—C19	123.6 (2)
C4—C3—H3	120.1	N4—C23—C24	115.89 (17)
C12—C4—C3	116.4 (2)	C19—C23—C24	120.52 (19)
C12—C4—C5	118.1 (2)	N3—C24—C16	123.66 (19)
C3—C4—C5	125.6 (2)	N3—C24—C23	115.81 (17)
C6—C5—C4	121.4 (2)	C16—C24—C23	120.53 (18)
C6—C5—H5	119.3	O3—C25—O2	124.8 (2)
C4—C5—H5	119.3	O3—C25—O1	124.9 (2)
C5—C6—C7	121.3 (2)	O2—C25—O1	110.31 (19)
C5—C6—H6	119.4	O3—C25—Co1	179.7 (2)
C7—C6—H6	119.4	O2—C25—Co1	55.07 (10)
C11—C7—C8	117.0 (2)	O1—C25—Co1	55.24 (10)
C11—C7—C6	118.0 (2)	O4—N5—O5	122.1 (3)
C8—C7—C6	125.0 (2)	O4—N5—O6	119.6 (3)
C9—C8—C7	119.3 (2)	O5—N5—O6	118.3 (2)
C9—C8—H8	120.3	C1—N1—C12	118.63 (19)
C7—C8—H8	120.3	C1—N1—Co1	129.21 (16)
C8—C9—C10	120.3 (2)	C12—N1—Co1	112.16 (13)
C8—C9—H9	119.9	C10—N2—C11	117.83 (18)
C10—C9—H9	119.9	C10—N2—Co1	130.49 (15)
N2—C10—C9	122.0 (2)	C11—N2—Co1	111.66 (13)
N2—C10—H10	119.0	C13—N3—C24	117.73 (18)
C9—C10—H10	119.0	C13—N3—Co1	130.61 (14)
N2—C11—C7	123.53 (19)	C24—N3—Co1	111.66 (13)
N2—C11—C12	115.83 (18)	C22—N4—C23	118.39 (18)
C7—C11—C12	120.62 (19)	C22—N4—Co1	129.10 (15)
N1—C12—C4	123.3 (2)	C23—N4—Co1	112.51 (14)
N1—C12—C11	116.12 (17)	C25—O1—Co1	89.92 (13)
C4—C12—C11	120.5 (2)	C25—O2—Co1	90.28 (13)
N3—C13—C14	122.3 (2)	H7A—O7—H7B	105 (3)
N3—C13—H13	118.8	O2—Co1—O1	69.48 (7)
C14—C13—H13	118.8	O2—Co1—N4	92.34 (7)
C15—C14—C13	120.0 (2)	O1—Co1—N4	90.80 (7)
C15—C14—H14	120.0	O2—Co1—N1	91.02 (7)
C13—C14—H14	120.0	O1—Co1—N1	91.88 (7)
C14—C15—C16	119.5 (2)	N4—Co1—N1	176.28 (7)
C14—C15—H15	120.3	O2—Co1—N2	98.32 (7)
C16—C15—H15	120.3	O1—Co1—N2	167.16 (7)
C24—C16—C15	116.8 (2)	N4—Co1—N2	93.79 (7)
C24—C16—C17	118.0 (2)	N1—Co1—N2	84.13 (7)
C15—C16—C17	125.2 (2)	O2—Co1—N3	167.90 (7)
C18—C17—C16	121.4 (2)	O1—Co1—N3	98.94 (7)
C18—C17—H17	119.3	N4—Co1—N3	84.09 (7)
C16—C17—H17	119.3	N1—Co1—N3	92.94 (7)
C17—C18—C19	121.5 (2)	N2—Co1—N3	93.46 (7)
C17—C18—H18	119.3	O2—Co1—C25	34.65 (8)
C19—C18—H18	119.3	O1—Co1—C25	34.84 (8)
C23—C19—C20	116.7 (2)	N4—Co1—C25	91.74 (8)
C23—C19—C18	118.0 (2)	N1—Co1—C25	91.94 (8)
C20—C19—C18	125.2 (2)	N2—Co1—C25	132.87 (8)
C21—C20—C19	119.7 (2)	N3—Co1—C25	133.67 (8)
C21—C20—H20	120.2		
			
N1—C1—C2—C3	−2.1 (4)	C24—C23—N4—C22	−178.0 (2)
C1—C2—C3—C4	2.1 (4)	C19—C23—N4—Co1	−179.14 (17)
C2—C3—C4—C12	−0.4 (3)	C24—C23—N4—Co1	1.4 (2)
C2—C3—C4—C5	178.2 (2)	O3—C25—O1—Co1	−179.7 (2)
C12—C4—C5—C6	3.4 (3)	O2—C25—O1—Co1	−0.44 (17)
C3—C4—C5—C6	−175.2 (2)	O3—C25—O2—Co1	179.7 (2)
C4—C5—C6—C7	−2.6 (4)	O1—C25—O2—Co1	0.44 (17)
C5—C6—C7—C11	−0.3 (3)	C25—O2—Co1—O1	−0.31 (12)
C5—C6—C7—C8	179.3 (2)	C25—O2—Co1—N4	89.67 (13)
C11—C7—C8—C9	−0.1 (3)	C25—O2—Co1—N1	−91.93 (13)
C6—C7—C8—C9	−179.7 (2)	C25—O2—Co1—N2	−176.15 (12)
C7—C8—C9—C10	−1.0 (4)	C25—O2—Co1—N3	17.2 (4)
C8—C9—C10—N2	1.3 (4)	C25—O1—Co1—O2	0.30 (12)
C8—C7—C11—N2	1.2 (3)	C25—O1—Co1—N4	−91.89 (12)
C6—C7—C11—N2	−179.23 (19)	C25—O1—Co1—N1	90.69 (13)
C8—C7—C11—C12	−177.2 (2)	C25—O1—Co1—N2	19.1 (4)
C6—C7—C11—C12	2.4 (3)	C25—O1—Co1—N3	−176.04 (12)
C3—C4—C12—N1	−1.6 (3)	C22—N4—Co1—O2	10.6 (2)
C5—C4—C12—N1	179.7 (2)	C23—N4—Co1—O2	−168.73 (15)
C3—C4—C12—C11	177.4 (2)	C22—N4—Co1—O1	80.1 (2)
C5—C4—C12—C11	−1.3 (3)	C23—N4—Co1—O1	−99.24 (15)
N2—C11—C12—N1	−1.0 (3)	C22—N4—Co1—N2	−87.9 (2)
C7—C11—C12—N1	177.51 (18)	C23—N4—Co1—N2	92.76 (15)
N2—C11—C12—C4	179.92 (18)	C22—N4—Co1—N3	179.0 (2)
C7—C11—C12—C4	−1.6 (3)	C23—N4—Co1—N3	−0.33 (15)
N3—C13—C14—C15	0.0 (4)	C22—N4—Co1—C25	45.3 (2)
C13—C14—C15—C16	0.5 (4)	C23—N4—Co1—C25	−134.07 (16)
C14—C15—C16—C24	0.0 (3)	C1—N1—Co1—O2	79.12 (19)
C14—C15—C16—C17	178.2 (2)	C12—N1—Co1—O2	−101.04 (14)
C24—C16—C17—C18	0.8 (4)	C1—N1—Co1—O1	9.62 (19)
C15—C16—C17—C18	−177.4 (3)	C12—N1—Co1—O1	−170.55 (14)
C16—C17—C18—C19	−1.4 (4)	C1—N1—Co1—N2	177.38 (19)
C17—C18—C19—C23	0.4 (4)	C12—N1—Co1—N2	−2.78 (14)
C17—C18—C19—C20	−177.3 (3)	C1—N1—Co1—N3	−89.44 (19)
C23—C19—C20—C21	−0.8 (4)	C12—N1—Co1—N3	90.40 (14)
C18—C19—C20—C21	176.9 (3)	C1—N1—Co1—C25	44.5 (2)
C19—C20—C21—C22	1.0 (4)	C12—N1—Co1—C25	−135.69 (15)
C20—C21—C22—N4	0.0 (4)	C10—N2—Co1—O2	−86.24 (18)
C20—C19—C23—N4	−0.4 (3)	C11—N2—Co1—O2	92.43 (13)
C18—C19—C23—N4	−178.3 (2)	C10—N2—Co1—O1	−104.0 (3)
C20—C19—C23—C24	179.0 (2)	C11—N2—Co1—O1	74.6 (3)
C18—C19—C23—C24	1.1 (3)	C10—N2—Co1—N4	6.68 (19)
C15—C16—C24—N3	−1.0 (3)	C11—N2—Co1—N4	−174.65 (13)
C17—C16—C24—N3	−179.3 (2)	C10—N2—Co1—N1	−176.42 (19)
C15—C16—C24—C23	179.0 (2)	C11—N2—Co1—N1	2.25 (13)
C17—C16—C24—C23	0.7 (3)	C10—N2—Co1—N3	90.98 (18)
N4—C23—C24—N3	−2.2 (3)	C11—N2—Co1—N3	−90.35 (13)
C19—C23—C24—N3	178.36 (19)	C10—N2—Co1—C25	−89.2 (2)
N4—C23—C24—C16	177.82 (19)	C11—N2—Co1—C25	89.44 (16)
C19—C23—C24—C16	−1.7 (3)	C13—N3—Co1—O2	−107.1 (3)
C2—C1—N1—C12	0.1 (3)	C24—N3—Co1—O2	72.5 (3)
C2—C1—N1—Co1	179.97 (17)	C13—N3—Co1—O1	−90.6 (2)
C4—C12—N1—C1	1.7 (3)	C24—N3—Co1—O1	89.07 (14)
C11—C12—N1—C1	−177.32 (18)	C13—N3—Co1—N4	179.5 (2)
C4—C12—N1—Co1	−178.11 (16)	C24—N3—Co1—N4	−0.81 (14)
C11—C12—N1—Co1	2.8 (2)	C13—N3—Co1—N1	1.8 (2)
C9—C10—N2—C11	−0.3 (3)	C24—N3—Co1—N1	−178.57 (14)
C9—C10—N2—Co1	178.26 (16)	C13—N3—Co1—N2	86.1 (2)
C7—C11—N2—C10	−0.9 (3)	C24—N3—Co1—N2	−94.28 (14)
C12—C11—N2—C10	177.51 (18)	C13—N3—Co1—C25	−93.7 (2)
C7—C11—N2—Co1	−179.79 (16)	C24—N3—Co1—C25	85.94 (16)
C12—C11—N2—Co1	−1.3 (2)	O1—C25—Co1—O2	−179.50 (19)
C14—C13—N3—C24	−1.0 (3)	O2—C25—Co1—O1	179.50 (19)
C14—C13—N3—Co1	178.63 (17)	O2—C25—Co1—N4	−91.60 (12)
C16—C24—N3—C13	1.5 (3)	O1—C25—Co1—N4	88.90 (12)
C23—C24—N3—C13	−178.5 (2)	O2—C25—Co1—N1	88.99 (12)
C16—C24—N3—Co1	−178.18 (17)	O1—C25—Co1—N1	−90.51 (12)
C23—C24—N3—Co1	1.8 (2)	O2—C25—Co1—N2	5.21 (17)
C21—C22—N4—C23	−1.2 (4)	O1—C25—Co1—N2	−174.29 (11)
C21—C22—N4—Co1	179.49 (19)	O2—C25—Co1—N3	−175.08 (10)
C19—C23—N4—C22	1.5 (3)	O1—C25—Co1—N3	5.42 (16)

### Hydrogen-bond geometry (Å, °)

**Table:**

*D*—H···*A*	*D*—H	H···*A*	*D*···*A*	*D*—H···*A*
O7—H7A···O5	0.86 (2)	1.98 (2)	2.830 (3)	168 (5)
O7—H7B···O3^i^	0.81 (5)	2.03 (5)	2.809 (4)	164 (6)
C3—H3···O3^ii^	0.93	2.54	3.362 (3)	148
C5—H5···O1^ii^	0.93	2.56	3.415 (3)	153
C8—H8···O7^iii^	0.93	2.31	3.130 (4)	146
C9—H9···O6^iv^	0.93	2.35	3.234 (3)	158
C15—H15···O6^v^	0.93	2.45	3.370 (3)	170
C17—H17···O4^v^	0.93	2.50	3.416 (3)	167
C1—H1···Cg1^vi^	0.93	2.92	3.705 (3)	143

Symmetry codes: (i) *x*+1, *y*, *z*; (ii) *x*+1/2, −*y*+1/2, *z*+1/2; (iii) −*x*+3/2, *y*+1/2, −*z*+3/2; (iv) −*x*+1, −*y*+1, −*z*+1; (v) −*x*+1, −*y*, −*z*+1; (vi) −*x*+1/2, *y*−1/2, −*z*+3/2.
